# Supplementary material for: Deletion of wfs1 Impairs Oligodendrocyte Precursor Cells Dorsal Distribution and Myelination Through the wfs1-hmgcs1 Axis in Zebrafish
Source: Biology (Basel). 2026 Aug 16;15(16):1402. doi: 10.3390/biology15161402 (PMC13510151; doi:10.3390/biology15161402)
Supplement: Supplementary file 1 [file biology-15-01402-s001.zip › File S1-Transcriptome sequencing data and differentially expressed genes (DEGs) in wfs1-- zebrafish at 5 dpf/2.KEGG/wfs1KOvsWT/ALL/src/dre00670.html]

dre00670


- dre:100004977

- Down regulated genes

100004977\_2(-3.12630634600606)

- dre:557552

- Up regulated genes

557552(1.27169280646928)

- dre:557552

- Up regulated genes

557552(1.27169280646928)

- dre:100004977

- Down regulated genes

100004977\_2(-3.12630634600606)

- dre:100526683

- Down regulated genes

100526683\_1(-1.80591024105284)

- dre:557552

- Up regulated genes

557552(1.27169280646928)

Close
